# Non-Contact Ultrasonic Assessment of Corrosion in Steel Specimens

**DOI:** 10.3390/s26123923

**Published:** 2026-06-20

**Authors:** Lukas Peterson, Andrei Zagrai, ThankGod Nwokocha, T. David Burleigh

**Affiliations:** 1 Mechanical Engineering Department, New Mexico Institute of Mining and Technology, 801 Leroy Pl, Socorro, NM 87801, USA; 2 Materials & Metallurgical Engineering, New Mexico Institute of Mining and Technology, 801 Leroy Pl, Socorro, NM 87801, USAthomas.burleigh@nmt.edu (T.D.B.)

**Keywords:** corrosion, EMAT, resonance, thickness measurement, liftoff, dispersion curve, steel

## Abstract

Ultrasonic thickness resonance can be effectively used to detect and quantify the level of corrosion in steel nuclear storage containers as well as other corrosion-prone thin-walled structures, such as pipes and storage tanks. Electro-Magnetic Acoustic Transducers (EMATs) have several advantages over more traditional piezoelectric-based transducers; namely, they can be used in a non-contact fashion on robotic platforms, allowing for measurements regardless of surface conditions or temperature. The major challenge of EMAT application is the power required to counteract the low actuation efficiency, which is achieved with a high-power ultrasonic pulse generator and a transformer circuit. Resonance techniques, in which most of the energy is concentrated near structural resonance frequencies, are preferable to improve efficiency of electro-magnetic acoustic measurements. This methodology was applied to 316L stainless steel thin plates subjected to uniform corrosion as well as pitting corrosion imitating different damage scenarios in a nuclear waste container. The resonant peak frequency shift was found to be proportional to the severity of corrosion for minimally corroded samples. However, the complete disappearance of the resonance peak was observed in the samples with severe corrosion damage. The EMAT liftoff distance was studied to quantify its effect on the amplitude, spread, and frequency of resonant peaks. Recommendations for use of EMATs for assessing corrosion damage are presented. The study demonstrates the success of frequency-based detection of corrosion damage in 316L stainless steel used in fabrication of nuclear waste storage containers.

## 1. Introduction

### General Corrosion Problem

Thin-walled structures are incredibly common in a variety of engineering applications, the most well-known among these are in the skins of aircraft and in fluid handling equipment, such as pipes and pressure vessels [1]. While thin-walled structures are efficient in terms of their use of structural material, they are also susceptible to damage, such as corrosion, scratches, and dents. This predisposition to damage makes regular inspection or replacement a necessity for thin-walled structures. While scratches and dents are identifiable with visual inspection, this study focuses on assessment of corrosion, which can develop in the inner structural surfaces and hence is only detectable using advanced non-destructive evaluation methods. A number of methods exist to monitor corrosion in structures using permanently installed sensors, such as piezoelectric wafer arrays. Similarly, fiber optic cables can be mounted to a corrosion-prone surface and be used to monitor corrosion using optical frequency domain reflectometry [2].

Corrosion occurs in many forms, but the forms that affect thin-walled structures the most are uniform corrosion and/or pitting. Uniform corrosion, as the name implies, removes material in an even and consistent manner and is most commonly caused by acidic solutions, whereas pitting, on the other hand, is typically caused by localized electro-chemical reactions leading to craters in a metal [3]. Inspection of corrosion damage in thin-walled structures is not trivial as normally only one side of a structure is accessible. A typical approach to detect corrosion damage is to measure the structural thickness and its reduction due to ongoing corrosion [4]. There exist several methods for obtaining a thickness map of thin-walled structures. Most prominently, laser depth mapping and ultrasonic methods are widely used [5,6,7,8,9,10]. The next major problem is that the accessible side of thin-walled structures are often painted to protect the structures from corrosion, as thin-walled structures can rapidly develop holes from corrosion, invalidating methods that do not directly inspect the metal, such as the previously mentioned depth mapping and many ultrasonic methods. A popular method used to ultrasonically measure the thickness of thin-walled structures covered in a protective coating is Electro-Magnetic Acoustic Transducers (EMATs). The majority of studies that have measured the thickness of thin-walled structures using EMATs have focused on cylindrical structures because the corrosion on the interior of a pipe is particularly troublesome for petroleum refining, chemical production and rocket motor casings [11]. Inspecting the group and phase velocities has the additional benefit of allowing for the determination of stress in thin-walled structures [12]. Many guided wave studies conducted in the last 5 to 10 years have used broad-band excitation and a Fast Fourier Transform (FFT) to extract the dispersion curve information [13,14,15]. This methodology significantly decreases the time required to generate dispersion curves, but it is often less precise and requires the use of higher-end data acquisition equipment. There has also been interest in using either phased arrays or moving transducers to create maps of damage location and intensity [16,17,18,19,20,21]. The studies that have used phased arrays typically use piezoelectric wafers as transducers and often use either machine learning or tomography to perform data processing. Many of these techniques have trouble detecting small changes in exceptionally thin plates, as is the case with mild cases of corrosion.

The majority of prior work on corrosion detection and severity determination using Electro-Magnetic Acoustic Transducers (EMATs) was done in the 1990s by M. Gori et al. using a guided wave phase delay for boilers [22,23]. Since the 1990s there has been sporadic work on using EMATs to detect corrosion, despite commercial systems being available [24,25,26]. More recent work has focused on detecting other types of damage using EMATs or on using different transducer types to detect corrosion. Wafer Active Sensors (PWAS), submersion transducers, and phased arrays of the previously mentioned transducers have become the transducers of choice recently for many applications [27,28].

Corrosion has long been a problem in storage containers for compounds related to the nuclear industry. Corrosion is usually induced by radiation, the result of strong acids, or a result of off-gassing. Thus, the important types of corrosion that must be monitored and inspected for are stress corrosion cracking, crevice corrosion, pitting corrosion, uniform corrosion, high-temperature radiation, and radiation-induced corrosion [29,30,31,32,33,34,35]. Significant resources have been put into studying the corrosion process, as well as monitoring such containers. Corrosion monitoring research has included neural network classification of optical images, eddy current monitoring, and the use of ultrasonic waves to verify material dimensions [36,37,38,39]. However, all these studies required firm contact with the container and an intricate ultrasonic setup at 20 MHz frequencies. The significance of this work is that it proposes a resonant frequency approach to determine the thickness of 316L stainless steel containers used for the storage of nuclear compounds and waste. In contrast to other methods, such an approach provides a rather fast and potentially non-contact methodology for assessing corrosion damage. A considerable advantage of this method is its straightforward integration with robotic inspection platforms.

## 2. Guided Waves and Cutoff Resonances

### Guided Waves

The speed at which an ultrasonic wave travels through a thin plate is dependent on the plate’s thickness, frequency, and material properties. Lamb waves have particle motion in the directions of propagation and thickness (particle movement in the X and Z axes). Because of the dynamics in the thickness direction, Lamb waves are dispersive, which means that their propagation speed depends on spectral characteristics of the propagating wave. Because of this, Lamb waves were chosen to monitor reductions in thickness due to corrosion. The phase velocity dispersion curves can be calculated using Equation (1) for anti-symmetric (A) modes and Equation (2) for symmetric modes (S) for all prospective thicknesses [40].

(1) $$\frac{tan\;pd}{tan\;qd}=-\frac{{\left(\xi^{2}-q^{2}\right)}^{2}}{4\xi^{2}pq}$$

(2) $$\frac{tan\;pd}{tan\;qd}=-\frac{4\xi^{2}pq}{{\left(\xi^{2}-q^{2}\right)}^{2}}$$

(3) $$p^{2}=\frac{\omega^{2}}{C_{p}^{2}}-\xi^{2}$$

(4) $$q^{2}=\frac{\omega^{2}}{C_{s}^{2}}-\xi^{2}$$

**Figure 1:**
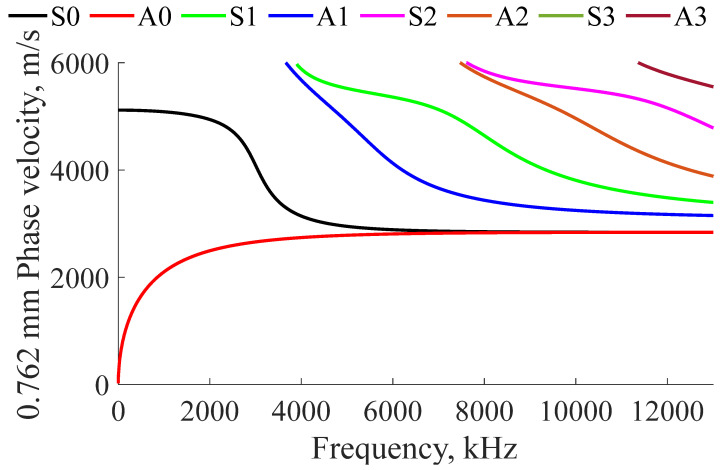
Analytical Lamb phase velocity in 0.762 mm thick 316L stainless steel plate.

**Figure 2:**
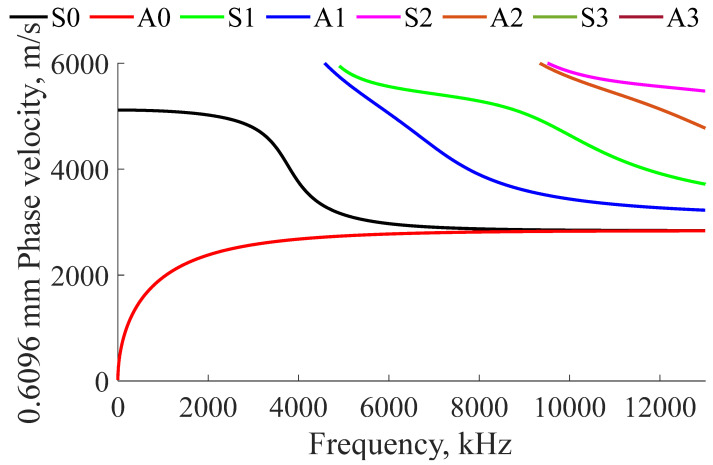
Analytical Lamb phase velocity in 0.6096 mm thick 316L stainless steel plate.

For Equations (1)–(4) ξ is the wavenumber, ω is the frequency (rad/s), $C_{p}$ is the phase velocity (m/s), and $C_{g}$ is the group velocity (m/s). p and q are defined by Equations (3) and (4), respectively. Examples of Lamb wave phase velocity dispersion curves calculated for 316L stainless steel plates with thicknesses closely resembling the thicknesses of storage containers are presented in Figure 1 and Figure 2. Using the phase velocity data, the group velocity plots can be found using Equation (5), where f is the frequency (Hz) and d is the plate thickness (m).

(5) $$C_{g}=C_{p}^{2}{\left(C_{p}-fd\frac{\partial C_{p}}{\partial (fd)}\right)}^{-1}$$

**Figure 3:**
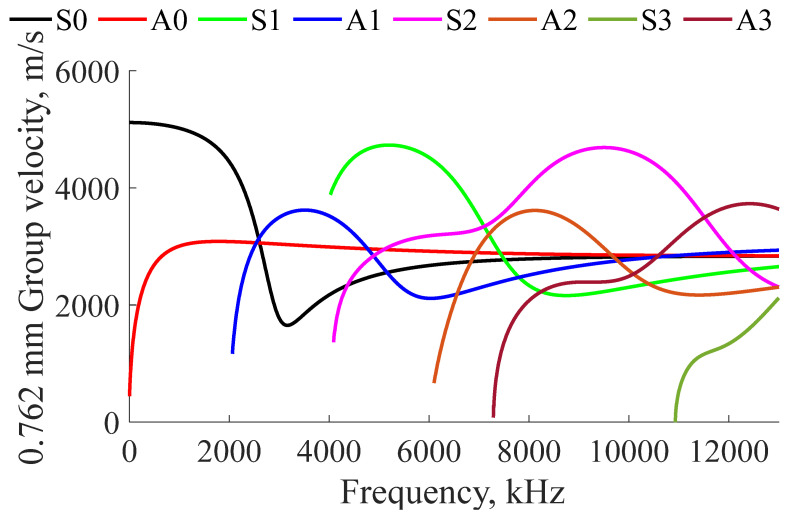
Analytical Lamb group velocity in 0.762 mm thick 316L stainless steel plate.

**Figure 4:**
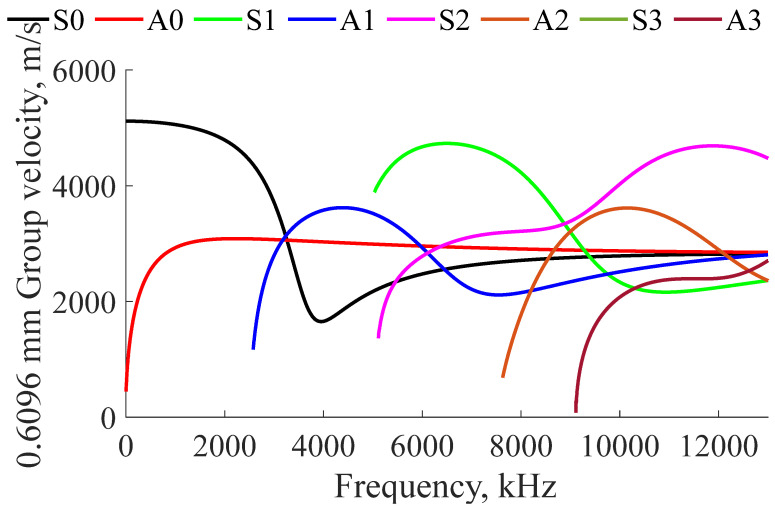
Analytical Lamb group velocity in 0.6096 mm thick 316L stainless steel plate.

Because the continuous derivative of the phase velocity is difficult to calculate, the finite difference approximation is used, as seen in Equation (6). The result of utilizing this approximation can be seen in Figure 3 and Figure 4.

(6) $$C_{g}\approx C_{p}^{2}{\left(C_{p}-fd\frac{\Delta C_{p}}{\Delta (fd)}\right)}^{-1}$$

Examples of Lamb wave group velocity dispersion curves calculated for 316L stainless steel plates with thicknesses closely resembling the thicknesses of storage containers are presented in Figure 3 and Figure 4. These plots are asymptotes of the finite difference approximation, which occasionally cause instabilities at extreme points when the curves approach vertical lines. These asymptotes are where resonant frequencies will appear.

**Figure 5:**
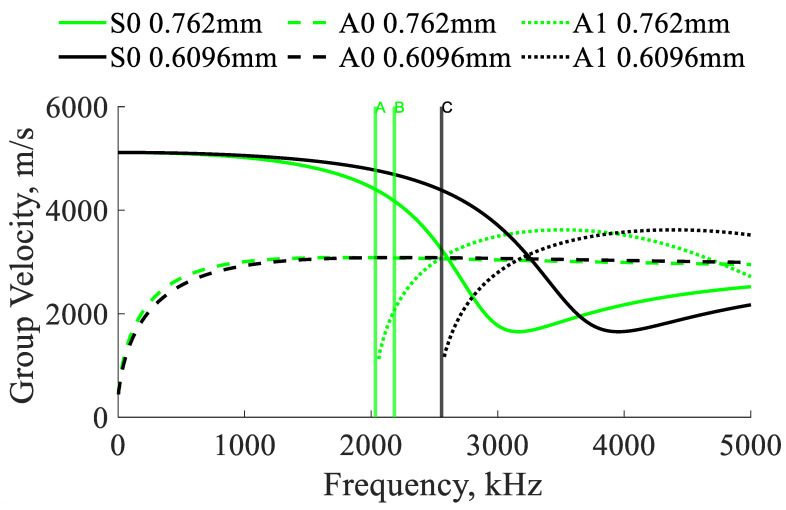
Analytical and experimental Lamb group velocities for two stainless steel plates. A: Experimental resonance of 610 mm by 610 mm by 0.762 mm sample; B: experimental resonance of 305 mm by 127 mm by 0.762 mm plate; and C: experimental resonance of a 610 by 610 mm by 0.6096 mm plate [42].

A comparison of group velocities for two thicknesses of stainless steel plates are presented in Figure 5. The asymptotic vertical lines occur at the thickness resonant frequencies [41]. As the thicknesses of samples change due to corrosion, it is anticipated that the vertical asymptotes, and thus the resonant frequencies, will shift, enabling the detection of corrosion damage.

## 3. Experimental Materials, Methods, and Algorithms

### 3.1. Electro-Magnetic Acoustic Transducers

EMATs were selected, as opposed to other ultrasonic transducers, due to their ability to operate in a non-contact fashion. Other transducers often require the use of a bonding agent, making them suboptimal for automated operation. EMATs operate using one of several different operating principals depending on the material. EMATs operating on ferromagnetic samples use a combination of the magnetostriction effect and the Lorentz force, while EMATs operating on non-ferromagnetic samples use only the Lorentz force. EMATs operating on ferromagnetic materials primarily rely on the magnetostriction effect as the Lorentz force is much lower in amplitude. Magnetostriction is a strain induced by a changing magnetic field on the surface of a material. EMATs are capable of receiving signals because the changing magnetic field in steel caused by ultrasonic waves induces a small voltage inside the coil. The primary advantages offered by EMATs is the ability to operate in a non-contact fashion, allowing for fast scanning over sample surfaces.

**Figure 6:**
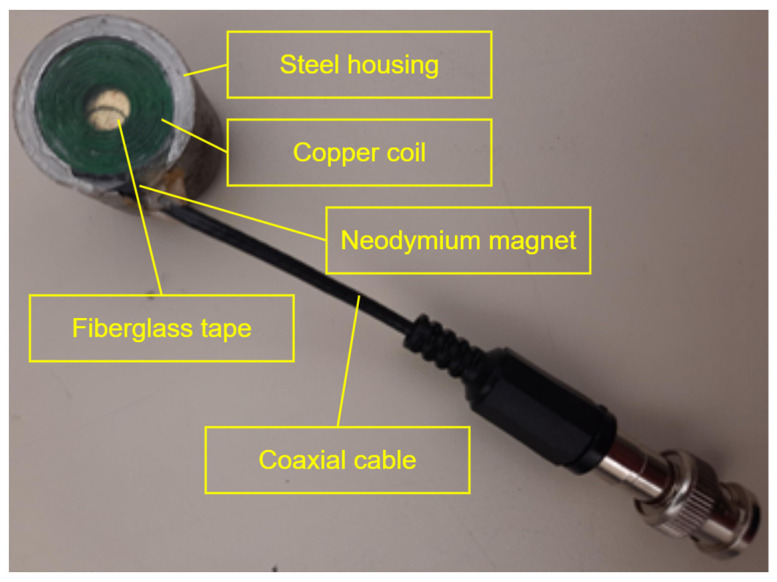
The steel housing, copper coil, neodymium magnet, and coaxial cable are the primary components of the custom laboratory-built EMATs.

The first EMAT employed in this paper used a 3/4rdx1/8 grade N52 magnet and a coil with an inner diameter of 0.25, an outer diameter of 0.75, a wire gauge of 30 (wire diameter of 0.011), 9 layers, and 205 turns. The second EMAT used a 3/4rdx1/8 grade N52 magnet and a coil with an inner diameter of 0.203, an outer diameter of 0.738, a wire gauge of 30 (wire diameter of 0.011), 9 layers, and 219 turns. The coil was built by winding an enamel-insulated magnet wire around a jig with the proper dimensions while liberally applying common epoxy to the wires to ensure the coils’ mechanical integrity. The components were then assembled inside a machined steel housing by first adding a neodymium magnet, followed by fiberglass carpet tape, followed by the previously assembled coil, and finally the unused space inside the EMAT was filled with common epoxy [43]. The components inside the steel shielding can be seen Figure 6.

### 3.2. EMAR Methodology

**Figure 7:**
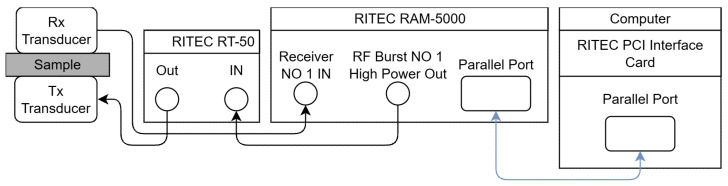
Experimental setup using custom EMATs, RITEC RAM-5000, RITEC control computer, and RITEC RT-50 attenuator. Note that arrows indicate the directions that signals travel.

For the Electro-Magnetic Acoustic Resonance (EMAR) experiments, the first EMAT was placed on top of the sample while the other EMAT transducer was placed below the sample, as depicted in Figure 7. Proper alignment between the EMATs was ensured by the neodymium magnets inside the EMATs. A RITEC RAM-5000, RITEC Inc., Warwick, RI, 02886, USA, was used to both generate the high-voltage signals and to record the returning signal. The sample was excited between 1.8 MHz and 2.4 MHz with an increment of 100 Hz. The frequency range was chosen to include the cutoff frequencies of the A1 mode for multiple plate thicknesses. The burst width was set at 200 µs with a burst delay of 1.6125 µs, and an output level of 80.

Once the signals returned to the RITEC, the amplifier was set at 40 dB, and a 1 MHz high-pass filter and 20 MHz low-pass filter were used to reduce the spurious components. A RITEC superheterodyne receiver was applied to improve signal reception. A superheterodyne receiver utilizes the results of mixing the amplified received signal with a locally generated reference signal (LO). The mixing results in a signal at intermediate frequency (IF), which is a bandwidth independent of the operation frequency and immune to out-of-band spurious signals. Once the signal is at this frequency, its phase angle and amplitude are found using quadrature phase-sensitive detectors. The RITEC RAM-5000 utilizes an I/Q receiver. A detailed operational diagram is available in [44].

**Figure 8:**
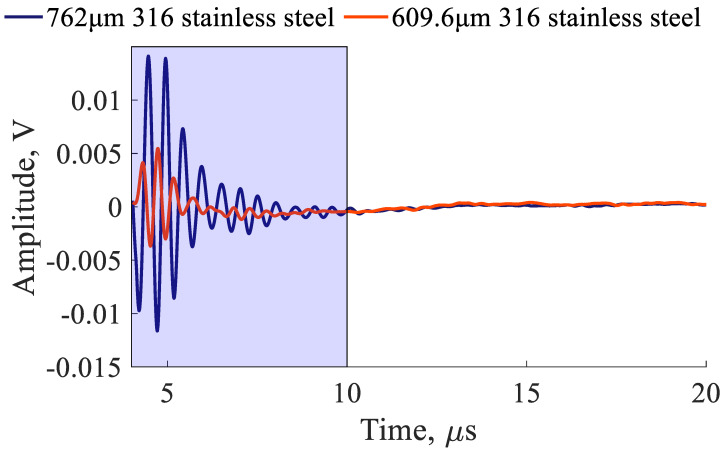
Example of EMAR signals with proper integration window for experiment involving plates of different thicknesses.

In an I/Q receiver, the incoming signal, represented by Equation (7), is split into orthogonal components with a 90° or $\frac{\pi }{2}$ radians offset, as represented by Equations (8) and (9), where $A_{r}\left(t\right)$ is the amplitude of incoming signals, $f_{r}$ is the characteristic incoming frequency, $\phi_{r}$ is the incoming signal phase offset, and $g_{3}$ is the total gain and conversion efficiencies of the phase detectors. To get a spectral characterization of the sample, the signal must be transformed into the frequency domain. Most modern systems utilize an FFT to represent signals in the frequency domain, but the RITEC RAM-5000 utilizes an integration window, as illustrated by Equations (10) and (11). Integration take place between two times, $t_{1}$ and $t_{2}$; the delayed start to the integration prevents interference from the transmitted signals while the end of the integration window minimizes extraneous data. An example of the integration window for the EMAR experiments with plates of different thicknesses can be seen in Figure 8. Once the orthogonal signals are integrated, they are measured using a 16-bit analog-to-digital converter. Using these two values, a value proportional to the impedance can be found using Equation (12). Similarly, if desired, the phase angle can also be reconstructed using Equation (13).

(7) $$f\left(t\right)=A_{r}\left(t\right)sin\left(2\pi f_{r}t+\phi_{r}\right)$$

(8) $$D_{1}\left(t\right)=g_{3}A_{r}\left(t\right)sin(\phi_{r})$$

(9) $$D_{2}\left(t\right)=g_{3}A_{r}\left(t\right)cos{(\phi }_{r})$$

(10) $$I_{1}=\int_{t_{1}}^{t_{2}}A\left(t\right)\cdot sin\left(\phi_{r}\right)dt$$

(11) $$I_{2}=\int_{t_{1}}^{t_{2}}A\left(t\right)\cdot cos\left(\phi_{r}\right)dt\;A$$

(12) $$A_{mag}=\sqrt{I_{1}^{2}+I_{2}^{2}}$$

(13) $$\phi_{r}={tan}^{-1}\left(\frac{I_{2}}{I_{1}}\right)$$

## 4. Ultrasonic Investigations of Intact (Uncorroded) Samples

### Resonance Measurement

**Figure 9:**
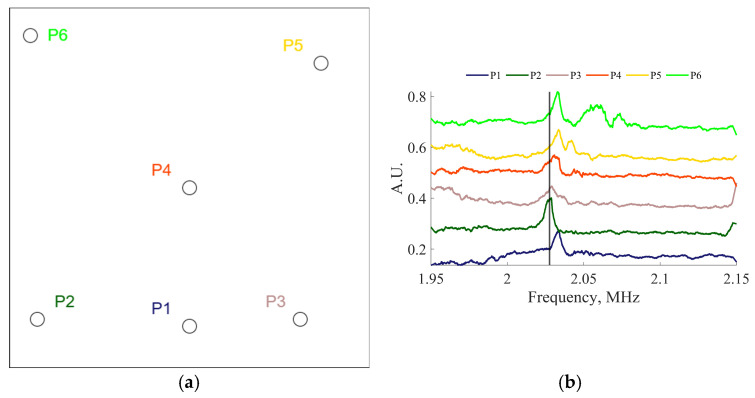
(**a**) Locations of resonance measurement. (**b**) Thickness resonances measured at various locations on a 610 mm by 610 mm by 0.762 mm steel plate.

The Electro-Magnetic Acoustic Resonance (EMAR) approach was studied to improve the sensitivity to thickness reduction due to corrosion and facilitate spatial mapping of structural thickness. The repeatability of the EMAR methodology, regardless of transducer location, was established. Two EMATs were placed below and above a 610 mm by 610 mm by 0.762 mm steel plate at six different random locations, labeled as P1 through P6, as depicted in Figure 9a. The experimental results are reported in Figure 9b, showing that the difference between the thickness resonance frequencies of 2.03 MHz measured at various locations was small and was around 5 kHz, which confirms repeatability of thickness assessment. At these frequencies it was anticipated that the wave propagation paths would stay primarily under the footprints of transducers, allowing for a concentration of elastic energy at resonance. Due to the low efficiency of EMATs operating on ferromagnetic materials, modes at frequencies higher than A1 were not observed.

## 5. Effect of Liftoff in Resonance Experiments

The EMAT efficiency decreases as the liftoff distance increases, and it is essential to explore this dependency. Analyzing the experimental results, it is possible to plot a curve for the observed signal strength versus liftoff distance.

**Figure 10:**
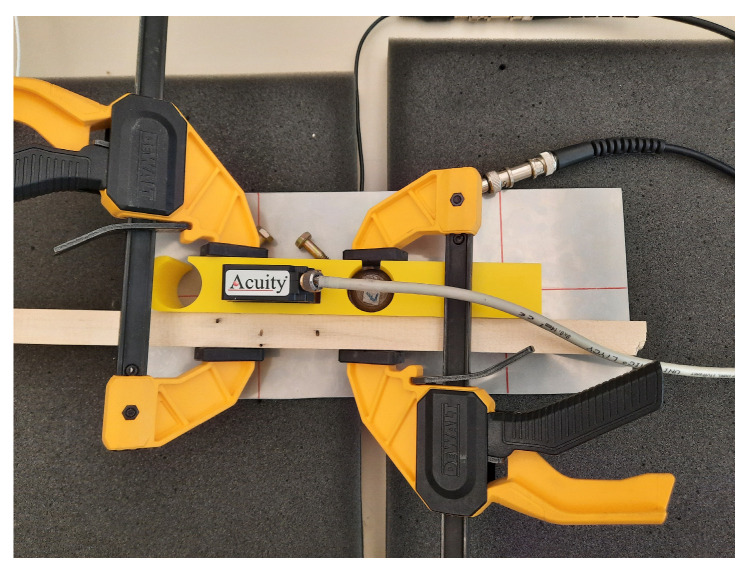
Experimental setup for liftoff experiments consisting of a pristine plate on foam blocks, with one EMAT in a 3D-printed jig containing a laser distance sensor. The other EMAT is below the plate and was aligned using the mutual magnetic attraction of the magnets in the EMATs. The wood and clamps were used to increase structural stiffness and ensure repeatable positioning.

**Figure 11:**
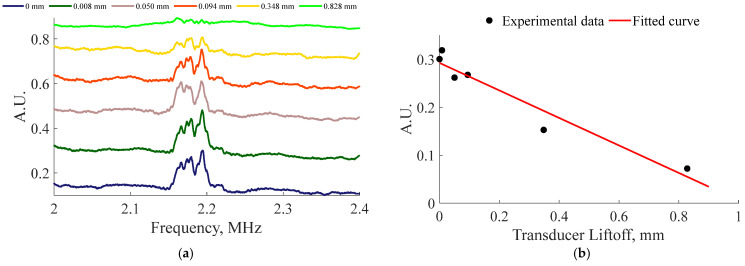
(**a**) Resonance spectra demonstrating the effect of EMAT liftoff on EMAR measurements. (**b**) Transducer liftoff versus received amplitude and a fitted curve.

In the EMAR liftoff tests, one of the EMATs was placed under the sample while the other EMAT was placed in a 3D-printed liftoff jig incorporating an Acuity A100 laser distance sensor to determine liftoff, as seen in Figure 10. The magnets contained within the EMATs were used to align the transducers. Starting at the lowest liftoff distance, resonance experiments were conducted between 0 and 1 mm without changing the EMAT location in relation to the plate. From the experimental results in Figure 11a, the amplitude was determined by measuring the maximum peak of EMAR amplitudes corresponding to the A1 mode. The equation describing the trend in Figure 11b is Amplitude=−0.2867×Liftoff+0.2924.

## 6. Corrosion Samples

To investigate the EMAR approach for the assessment of corrosion damage, a number of 316L stainless steel sheets, measuring 0.762 mm thick by 127 mm wide by 305 mm long, were exposed to 62.5 mL of different concentrations of HCl and FeCl_3_ solutions. All the samples were corroded at room temperature under different conditions. Prior to testing, the samples were cleaned with an alcohol reagent to remove dust or grease from the sample surfaces. There were two types of corrosion methods used for this study: the weight loss immersion test and the vapor test. Uniform corrosion, pitting corrosion and crevice corrosion were accelerated using a 50 mm diameter glass O-ring cylinder placed at the center of the stainless sheet with an O-ring between the stainless steel and the base of the glass cylinder. For the vapor corrosion samples, a 25 mL Erlenmeyer flask was placed on a stand in the center of the sheet, was filled with the acid of choice, and sealed. A larger 75 mm diameter glass O-ring cylinder tube was placed over the small flask. On the samples where crevice corrosion was undesirable, Apiezon L grease was applied generously around the O-ring and around the base of the glass cylinder to inhibit crevice corrosion. The cylinder was clamped tightly to the sheet with a steel clamp to prevent the solution from leaking out, while the top was sealed with Parafilm wax, preventing the escape of vapor. After accelerated corrosion was performed, the samples were washed using deionized water, cleaned with a plastic bristle brush, and the grease was removed with acetone.

**Figure 12:**
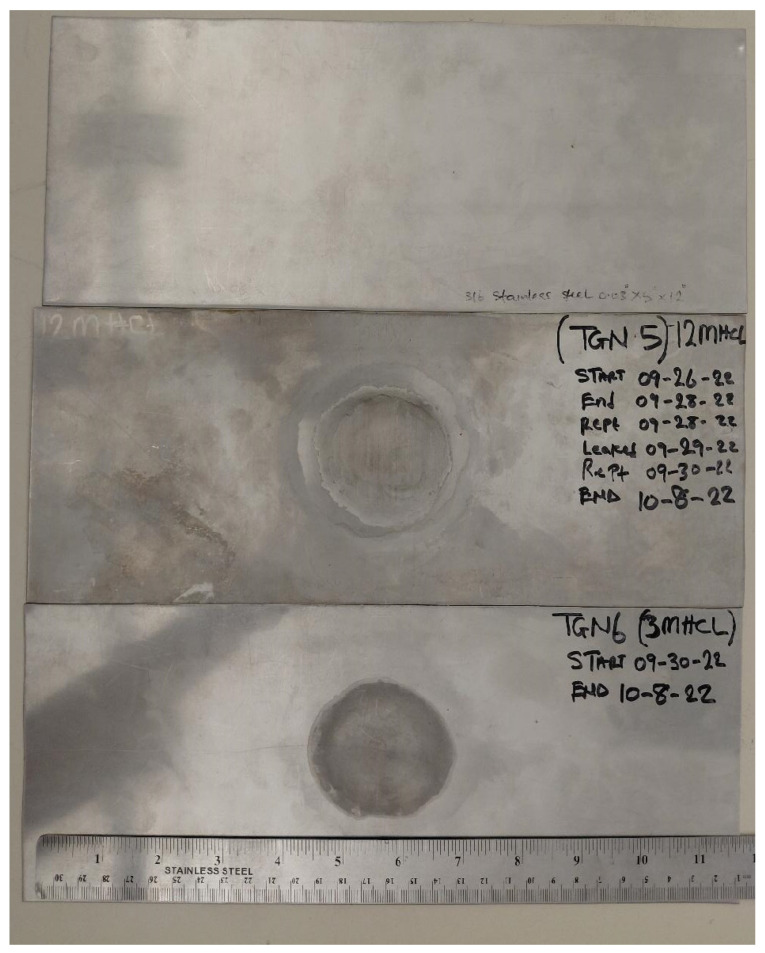
Pristine sample, TGN5 (12 M HCl for 11 days), and TGN6 (3 M HCl for 10 days).

**Figure 13:**
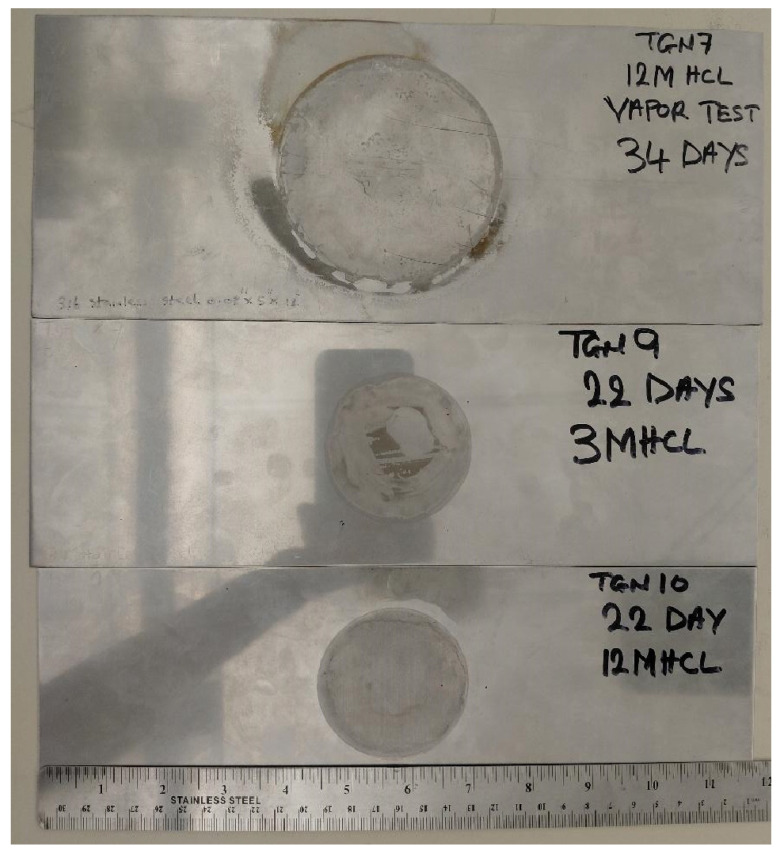
TGN7 (12 M HCl for 34 days), TGN9 (3 M HCl for 22 days), and TGN10 (12 M HCl for 22 days).

**Figure 14:**
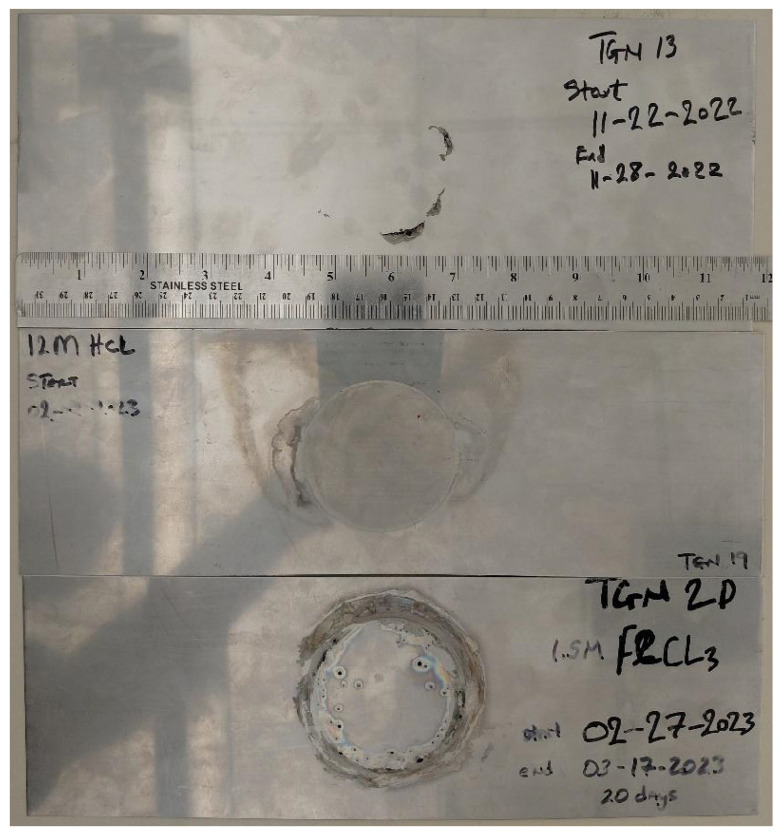
TGN13 (1.1 M FeCl_3_ for 6 days), TGN19 (12 M HCl for 14 days), and TGN20 (1.5 M FeCl_3_ for 20 days).

**Figure 15:**
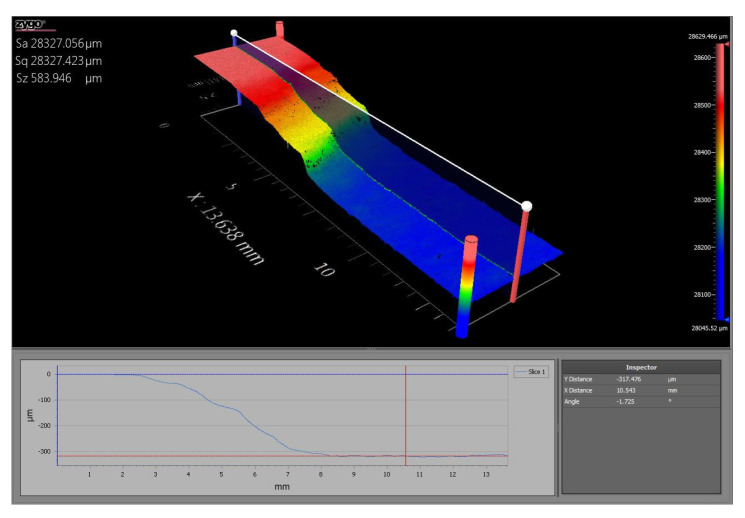
Laser profilometer results for TGN5 (12 M HCl for 11 days) showing a corrosion depth of 317.476 µm.

**Figure 16:**
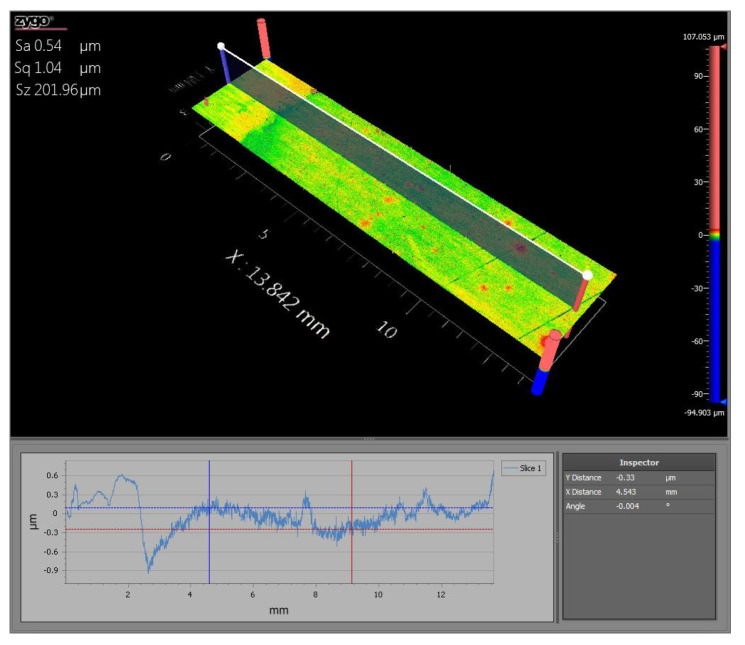
Laser profilometer results for TGN6 (3 M HCl for 10 days) showing a corrosion depth of 0.33 µm.

**Figure 17:**
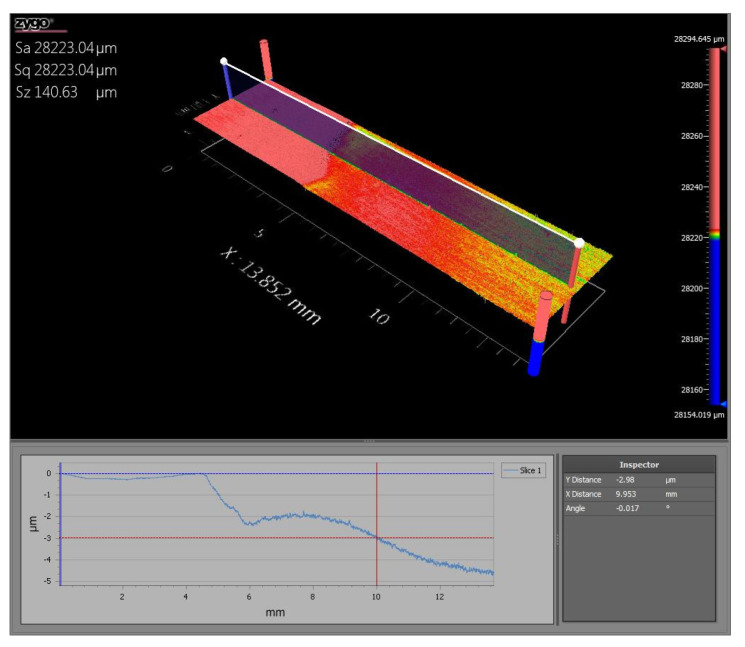
Laser profilometer results for TGN9 (3 M HCl for 22 days) showing a corrosion depth of 2.98 µm.

**Figure 18:**
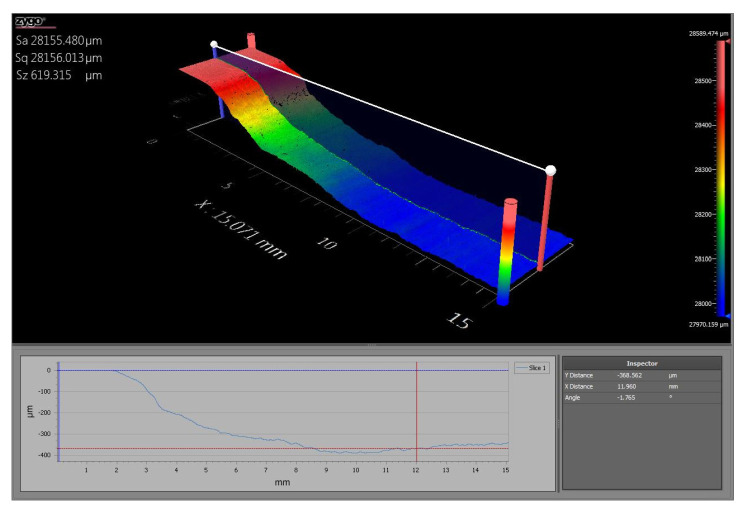
Laser profilometer results for TGN10 (12 HCl for 22 days) showing a corrosion depth of 368.562 µm.

**Figure 19:**
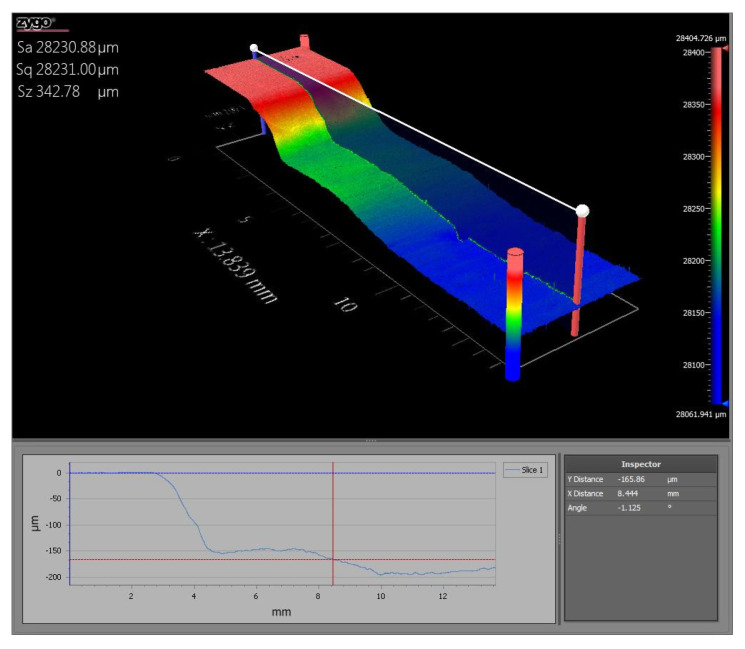
Laser profilometer results for TGN19 (12 M HCl for 14 days) showing a corrosion depth of 165.86 µm.

Note that a larger 75 mm diameter glass O-ring cylinder tube was used for sample TGN7. The actual corroded regions were larger than suggested by the size of the cylinder tube due to leaking. The acid used, corrosion type, exposure time, and corrosion depth can be found in Table 1. Photos of the corroded samples can be found in Figure 12, Figure 13 and Figure 14. The corrosion depth was measured using a laser profilometer as seen in Figure 15, Figure 16, Figure 17, Figure 18 and Figure 19; the values were found by taking the minimum thickness measured over the corroded region. Note that the corrosion depth of sample TGN7 was not measured as it was too large to fit in the laser profilometer. The samples with pitting and crevice corrosion could not be measured due to the uneven nature of the corroded surfaces.

## 7. Corrosion Monitoring Using the Resonance Approach

**Figure 20:**
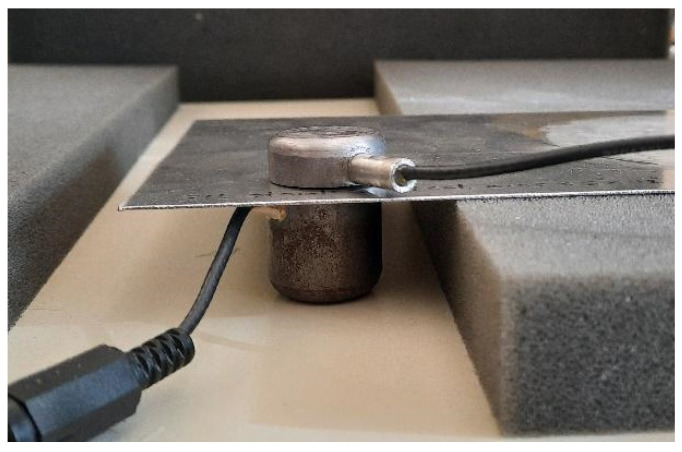
Experimental setup for resonance method.

In the resonance experiments, one EMAT was placed underneath each steel plate while another EMAT was placed on top of the plate, as illustrated in Figure 20, allowing for alignment of the EMATs to maximize detection signal. A pair of EMATs in this configuration were placed at semi-random locations on the plate. Boundary conditions were implemented by having a steel plate resting on foam blocks, as illustrated in Figure 20.

The RITEC RAM-5000 high-pass filter was set to 1 MHz, the low-pass filter was set to 20 MHz, and the amplifier was set to 40 dB. The gated integrator gate delay was set at 210 µs, the gate width was set at 50 µs, and the integration rate was set at 2000 V/Vms. A frequency scan was conducted between 1.8 MHz and 2.4 MHz with an increment of 100 Hz at a rate of 10 bursts/s. The output signal was set at a level of 80 with a burst width of 200 µs. Due to the initially noisy resonance data, a 101-point moving average was applied in postprocessing, yielding much cleaner spectra.

**Figure 21:**
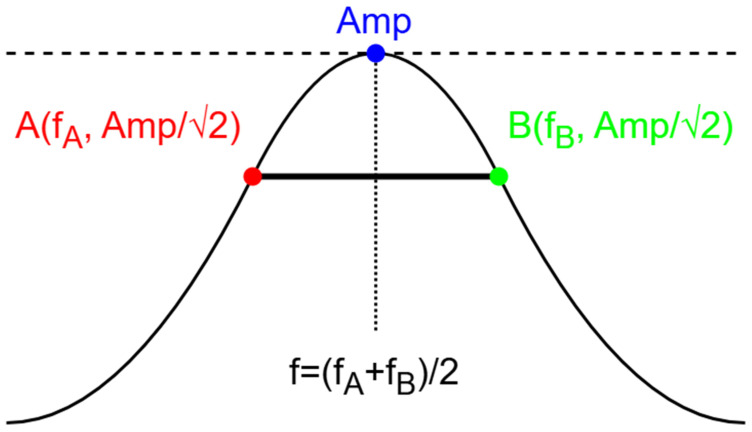
Diagram of peak-finding algorithm.

The middle of frequency peaks were found by taking the midpoint between the beginning and ending of the peak in question. The beginning and ending of peaks were defined as where the values dropped below the value of the peak divided by the square root of two, before and after the maximum of the peak. A diagram of this process can be found in Figure 21.

**Figure 22:**
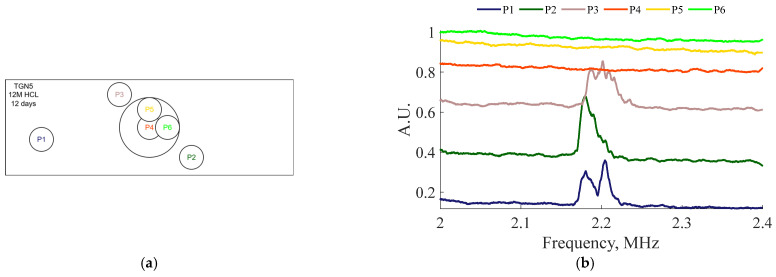
(**a**) Transducer locations for TGN5 (12 M HCl for 12 days). (**b**) Resonance results for TGN5 (12 M HCL for 12 days). Note that the resonance peaks disappear over the corroded regions.

**Figure 23:**
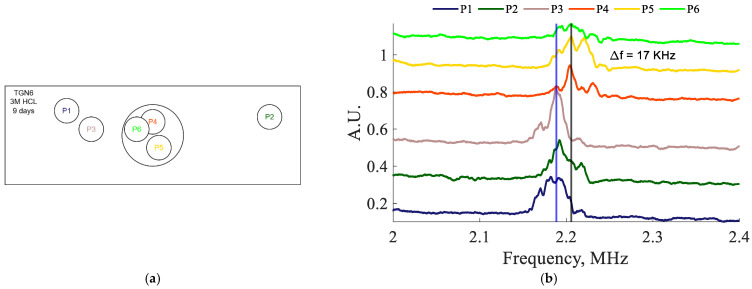
(**a**) Transducer locations for TGN6 (3 M HCl for 9 days). (**b**) Resonance test results for TGN6 sample showing shift in average frequency of 17 KHz. Note that the blue vertical line represents the median frequency peek of the experiments over the pristine region while the black vertical line represents the median peak frequency over the corroded region.

**Figure 24:**
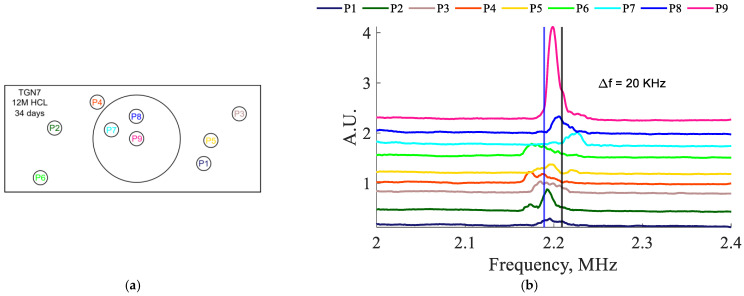
(**a**) Transducer locations for TGN7 (12 M HCl vapor for 34 days). (**b**) Resonance test results for TGN7 sample showing shift in average frequency of 20 KHz. Note that the blue vertical line represents the median frequency peek of the experiments over the pristine region while the black vertical line represents the median peak frequency over the corroded region.

**Figure 25:**
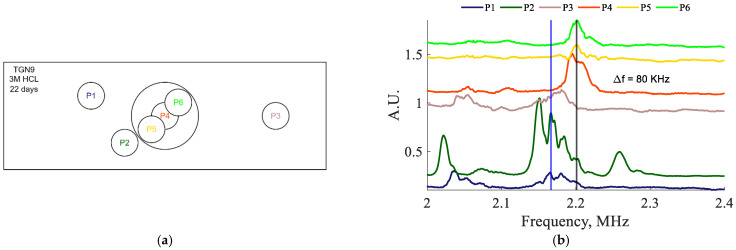
(**a**) Transducer locations for TGN9 (3 M HCl for 22 days). (**b**) Resonance test results for TGN9 sample showing shift in average frequency of 80 KHz. Note that the blue vertical line represents the median frequency peek of the experiments over the pristine region while the black vertical line represents the median peak frequency over the corroded region.

**Figure 26:**
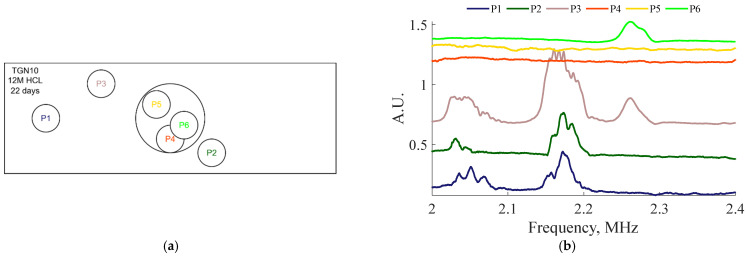
(**a**) Transducer locations for TGN10 (12 M HCl for 22 days). (**b**) Resonance results for TGN10.

**Figure 27:**
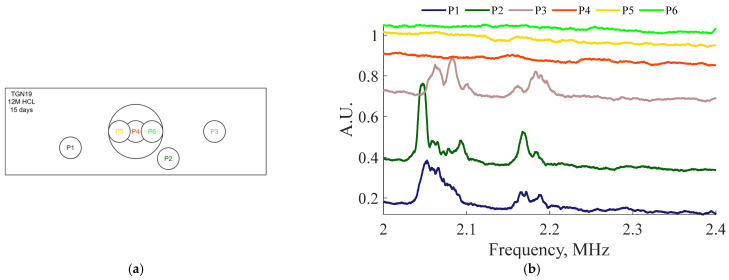
(**a**) Transducer locations for TGN19 (12 M HCl for 15 days). (**b**) Resonance results for TGN19 (12 M HCl for 15 days).

**Figure 28:**
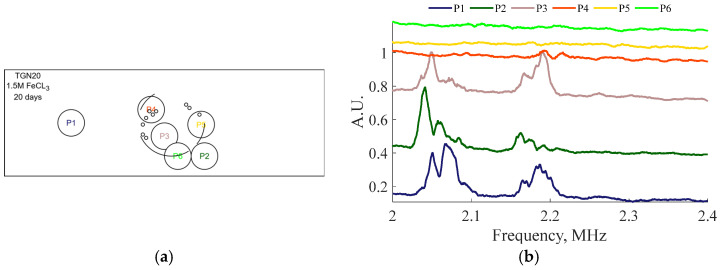
(**a**) Transducer locations for TGN20 (1.5 M FeCl_3_ for 20 days). (**b**) Resonance results for TGN20 (1.5 M FeCl_3_ for 20 days).

**Figure 29:**
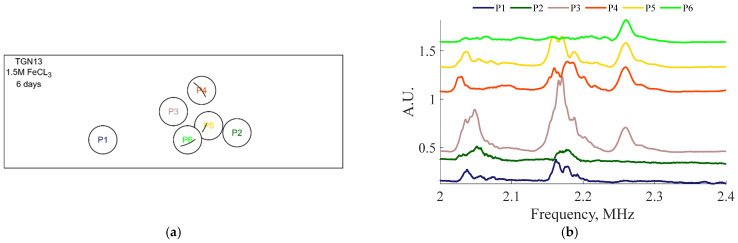
(**a**) Transducer locations for TGN13 (1.5 M FeCl_3_ for 6 days). (**b**) Resonance results for TGN13 (1.5 M FeCl_3_ for 6 days).

The results for the various samples can be seen in Figure 22, Figure 23, Figure 24, Figure 25, Figure 26, Figure 27, Figure 28 and Figure 29. The spectral results can be found in part (b) of the respective figures, while the measurement locations are represented in part (a) of the figures. Locations 1 through 3 (P1–P3) are located over pristine parts of the plates while the remaining three points are over the corroded regions of the plates (P4–P6). The results for TGN7, in Figure 24, includes an additional three pristine resonance results (P1–P6), while maintaining the standard three results from the corroded regions (P7–P9).

There is a small but recognizable tendency for the peaks to shift to a higher frequency; this is exemplified in TGN6 with a shift of 17 kHz, and in TGN7 with a shift of 20 kHz. This shift must be considered in light of measurement repeatability for each location on the plate. Should this technique be deployed, high measurement stability is recommended. The larger frequency shift in TGN7 correlates with the more pronounced corrosion damage in this sample. Of note is the impressive amplitude of the location 9 (P9) peak in the TGN7 experiment, due to its being located in the center of the corroded region. Where applicable, the blue vertical lines represent the average frequency peak over the pristine region while the black vertical lines represent the average frequency peak over the corroded regions.

The frequency shift present in Figure 23, Figure 24 and Figure 25 shows a correlation between the corrosion depth and the magnitude of frequency shift. In Figure 22, Figure 26, Figure 27 and Figure 28, the resonance frequencies disappear due to geometric misalignment of EMATs caused by corrosion. While quantification of corrosion is not possible in these cases, the qualitative assessment that corrosion is present is still useful. The results present in Figure 29 are uncertain because crevice corrosion is difficult to quantify with this technique.

**Figure 30:**
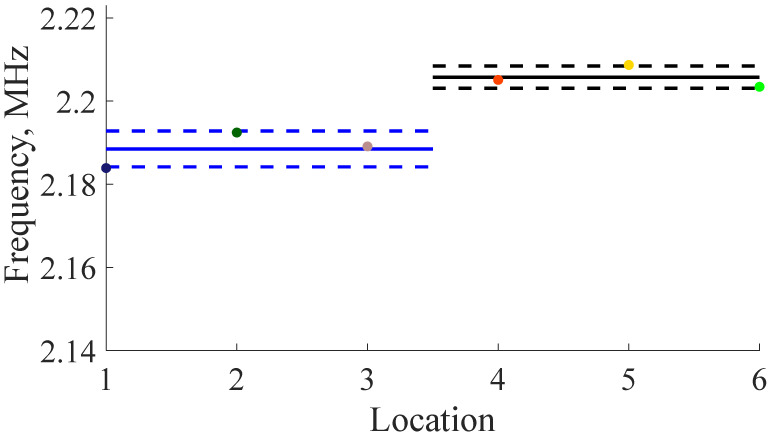
Plot showing frequency shift for all locations of TGN6 (3 M HCl for 9 days). Note that the solid blue line is the median frequency of the measurements over the pristine region while the solid black line the median of the measurements over the corroded region. The dotted lines show the bounds of one standard deviation.

**Figure 31:**
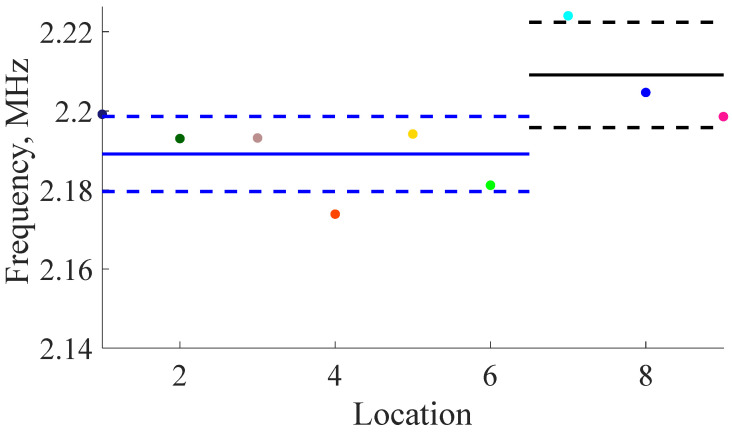
Plot showing frequency shift for all locations of TGN7 (12 M HCl vapor for 34 days). Note that the solid blue line is the median frequency of the measurements over the pristine region while the solid black line the median of the measurements over the corroded region. The dotted lines show the bounds of one standard deviation.

**Figure 32:**
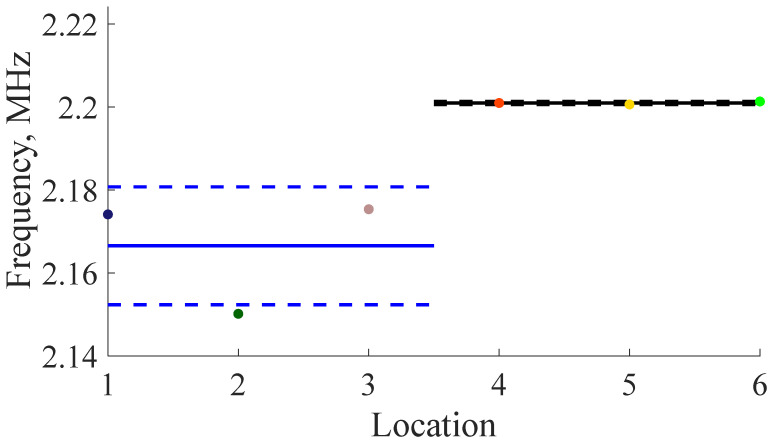
Plot showing frequency shift for all locations of TGN9 (3 M HCl for 22 days). Note that the solid blue line is the median frequency of the measurements over the pristine region while the solid black line the median of the measurements over the corroded region. The dotted lines show the bounds of one standard deviation.

**Figure 33:**
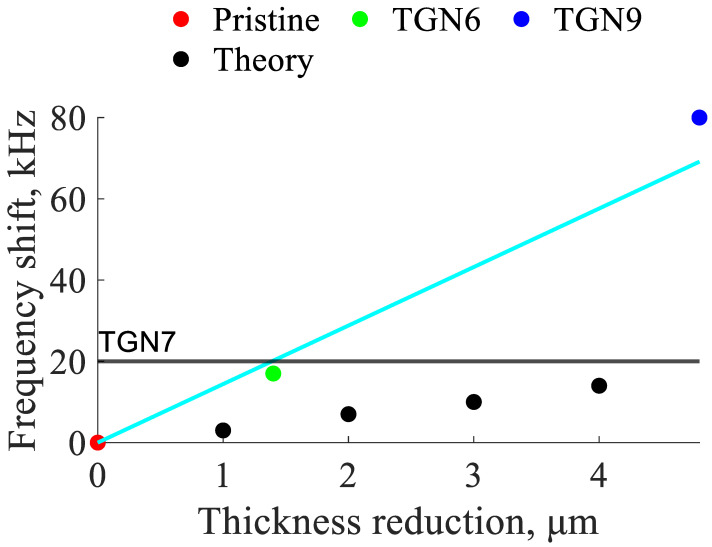
Plot showing mean frequency shift for pristine, TGN6 (3 M HCl for 9 days), TGN7 (12 M HCl vapor for 34 days), and TGN9 (3 M HCl for 22 days). The result for TGN7 is plotted as a horizontal line instead of a point because the thickness data is unavailable.

The shift in the average frequency of the A1 resonance can be seen in Figure 30, Figure 31 and Figure 32. While some level of averaging is necessary to obtain accurate measurements, it is possible to measure changes in thickness as small as 2 μm. A summary of the shift in the mean frequency and the associated change in sample thickness is presented in Figure 33. The theoretical frequency shift is 3.3333 kHz/µm while the experimentally measured frequency shift was found to be 14.4048 kHz/µm.

## 8. Conclusions

This work presents results of the thickness characterization of corroded metallic plates using the Electro-Magnetic Acoustic Resonance method. The resonant peak frequency shift was found to be proportional to the severity of corrosion for minimally corroded samples. It was found that by using averaging and an automatic peak-picking algorithm, it was possible to measure changes in thickness due to corrosion as small as 2 μm. For the samples with severe corrosion, the resonant peak disappeared due to the disintegrated material below the sensor that was not able to support generation of elastic waves at original frequencies, thus indicating that severe corrosion was present but not providing a measure of its severity. The effect of EMAT liftoff was also explored, showing significant loss of EMATs’ effectiveness when liftoff was larger than a millimeter. The significance of this work is that it demonstrates the success of frequency-based detection of corrosion damage in 316L stainless steel used in fabrication of nuclear waste storage containers. Due to its potential for non-contact operation, such technology could be directly integrated into robotic inspection platforms navigating in harsh environments. This will be a subject of future research.

## Figures and Tables

**Table 1:** Conditions used to produce corrosion samples. Note that “–“ indicates that the specific measurement was not taken.

Sample	Acid	Corrosion	Volume of Acid Used (mL)	Time (Days)	Sample Photo	Sample Thickness Over Corroded Region (mm)	Laser Profilometer Results
Pristine	N.A.	N.A.	N.A.	N.A.	Figure 12	0.762000	N.A.
TGN5	12 M HCl	Uniform 50 mm dia.	62 mL	11	Figure 12	0.441241	Figure 15
TGN6	3 M HCl	Uniform 50 mm dia.	62 mL	10	Figure 12	0.760580	Figure 16
TGN7	12 M HCl	Vapor 75 mm dia.	10 mL	34	Figure 13	-	-
TGN9	3 M HCl	Uniform 50 mm dia.	62 mL	22	Figure 13	0.757250	Figure 17
TGN10	12 M HCl	Uniform 24 mL dia.	62 mL	22	Figure 13	0.391992	Figure 18
TGN13	1.1 M FeCl_3_	Crevice/pitting 50 mm dia.	62 mL	6	Figure 14	N.A. due to pitting	-
TGN19	12 M HCl	Uniform 24 mm dia.	62 mL	14	Figure 14	0.57200	Figure 19
TGN20	1.5 M FeCl_3_	Pitting 24 mm dia.	62 mL	20	Figure 14	N.A due to pitting	-

## Data Availability

The availability of the data is subject to institutional and contractual policies. Please contact the corresponding author regarding data availability.
